# Nursing care for hospitalized patients with COVID-19 in light of Fundamental Care

**DOI:** 10.1590/0034-7167-2024-0075

**Published:** 2025-04-28

**Authors:** Fabieli Borges, Elizabeth Bernardino, Camila Rorato, Daniele Cristina dos Reis Bobrowec, Olívia Luciana dos Santos Silva, Amanda Gomes Ribeiro Pujol de Carvalho, Clémence Dallaire

**Affiliations:** I Universidade Federal do Paraná. Curitiba, Paraná, Brazil; II Université Laval. Quebec, Canada

**Keywords:** Nursing Assistance, Care, COVID-19, Patient, Hospital, Atención de Enfermería, Cuidado, COVID-19, Paciente, Hospital

## Abstract

**Objectives:**

to analyze how the COVID-19 pandemic affected the Fundamental Care provided by nurses to hospitalized patients in a public hospital.

**Methods:**

qualitative, descriptive, exploratory research. Twenty-four nurses were interviewed who cared for patients with COVID-19 in a public hospital in the capital city of the state of Paraná, from January to February 2022. Creswell Content Analysis was applied to the data, operationalized by the MaxQda software and in light of the Fundamental Care theory.

**Results:**

three categories were obtained with their respective dimensions: Physical Care (Personal Hygiene; Comfort and Mobilization; Eating and drinking; Rest and sleep; Safety and Medication Management), Psychosocial Care (Communication; Privacy; Dignity, respect and beliefs; Emotional well-being) and Relational Care (Active listening; Empathy and compassion; Engagement, support and involvement with families and caregivers and work with patients).

**Final Considerations:**

the pandemic period may have provided an opportunity to look at the nurse-patient-care relationship, especially based on the theory of Fundamental Care.

## RESUMEN

**Objetivos:**

analizar el impacto de la pandemia del COVID-19 sobre el Cuidado Fundamental de enfermería del paciente internado en un hospital público.

**Métodos:**

es una investigación cualitativa, descriptiva y exploratoria con 24 enfermeros entrevistados que cuidaron a pacientes con COVID-19 en un hospital público de la capital de Paraná, entre enero y febrero de 2022. Los datos se analizaron mediante el análisis de Contenido de Creswell con el *software* MaxQda bajo la luz de la teoría de los Cuidados Fundamentales.

**Resultados:**

surgieron tres categorías: Cuidados Físicos (Higiene Personal; Comodidad y Movilización; Comer y Beber; Descanso y Sueño; Seguridad y Gestión de la Medicación), Cuidados Psicosociales (Comunicación; Privacidad; Dignidad, Respeto y Creencias; Bienestar Emocional) y Cuidados Relacionales (Escucha Activa; Empatía y Compasión; Compromiso, Apoyo e Implicación de Familias y Cuidadores y, Trabajo con Pacientes).

**Consideraciones Finales:**

el periodo de pandemia ha permitido analizar la relación enfermero-paciente-atención bajo la teoría de los Cuidados Fundamentales.

## INTRODUCTION

The COVID-19 pandemic has presented an unprecedented challenge to the global population and to public health, even in high-income countries^(1,2)^. As part of the Health Care Network (HCN), teaching hospitals were requested by managers to provide ICU beds and specialized wards, so these facilities had to quickly implement structural care management and strategies to combat COVID-19^(1)^.

Due to the pressing need for hospital beds, the care provided to patients with COVID-19 has made the scheduling of the patient’s clinical conditions an essential criterion for making the best use of the care units that make up the HCN^(3)^. Based on what can be learned today and from the empirical observation shown daily about health scenarios, it is possible to reflect that the patient, in addition to the clinical issue imposed by the virus, may have been affected in other aspects of their health condition^(4,5)^.

Specifically during hospitalization, it is worth noting possible (lack of) nursing care for aspects related to the care provided to patients. In the meantime, a theoretical framework that supports the list of care proposed by Kitson and other authors a little over a decade ago, namely the so-called Fundamental Care (FC)^(6)^, can be used to shed light on the care at the time. Conceptually, FC refers to a holistic patient-centered care structure, encompassing nursing actions that respect and focus on a person’s essential needs to ensure their physical integrity and psychosocial well-being. Such needs are met through the development of a positive and trusting relationship with the person being cared for, as well as with their family and caregivers^(7,8)^.

Public teaching and federal hospitals sought to achieve their social responsibility in confronting the pandemic by valuing care, whether in direct care or in management actions. A documentary study conducted with 44 university hospitals in the federal education network in Brazil indicated, in the first year of the pandemic, a total of 495 actions to fight the COVID-19 pandemic, which were distributed among Assistance (38.99%), Management (37.58%), Outreach actions (16.16%) and Teaching and Research (7.27%)^(9)^. Since assistance is the action with the largest proportion, the relevance of this research is to understand how the provision of fundamental care occurred amid the demand experienced.

With all the structure and technology available, patients usually referred to hospitals were those who were most critical and dependent on care considered complex^(1,10)^. Although it was a challenge in terms of regulating beds and admission criteria for patients with COVID-19, when there was less supply than demand, this was added to the waiting lists, which previously were already a problem^(11-13)^.

The patient profile required a (re)orientation in the care provided by the nursing team at the bedside, especially with regard to Fundamental Care (FC). Based on this premise, the following guiding question is presented: how has the COVID-19 pandemic affected the provision of fundamental care by nurses in a public teaching hospital?

## OBJECTIVES

To analyze how the COVID-19 pandemic has impacted the Fundamental Care provided by nurses to hospitalized patients in a university hospital.

## METHODS

### Ethical aspects

This study was approved by the Ethics Committee of the *Hospital de Clínicas* Complex from the *Universidade Federal do Paraná*. Resolution 466 of the National Health Council of December 12, 2012 was respected. All participants voluntarily signed The Free and Informed Consent Form (FICF) presented at the beginning of this research. Furthermore, in order to respect the anonymity of the participants, the following coding was used: (Nurse) followed by the numerical order, Ex. Nurse 1, Nurse 2 and so on.

### Theoretical-methodological framework

This study is based on the theoretical framework of Fundamental Care (FC) proposed by Kitson and other authors^(6)^. Fundamental Care presents a structure that involves nurses’ actions in response to the person’s essential needs. It can be said, then, that fundamental care encompasses Physical Care activities, such as personal hygiene, comfort, rest and sleep; Psychosocial Care, encompassing privacy, dignity, emotional care and social interaction; and Relational Care, such as empathy, compassion and support^(7,8)^.

### Study Design

This is a qualitative, descriptive and exploratory research. The criteria presented in the Consolidated Criteria for Reporting Qualitative Research (COREQ) were followed^(14)^.

### Study Setting

The research was conducted at a public teaching hospital located in the capital of Paraná. The hospital is a reference for patients from all over the state, offering outpatient, clinical and surgical treatment, and it has around 424 beds. During the pandemic, the hospital expanded its capacity by adding 89 ward beds and 78 intensive care beds.

### Data Source

Nurses who worked directly in patient care during the COVID-19 pandemic, either during the day or the night, were included. Absence for the interview after three appointments agreed with the researcher, as well as the professionals’ vacation period or maternity leave were considered exclusion criteria for the research. The sample was intentional in order to select key representatives who worked on the front line during the pandemic. The nursing coordination of the infectious diseases unit, a central sector for meeting the demand of the COVID-19 pandemic in the institution, was initially contacted. The objectives of the study were explained and the interview was scheduled at the location, date and time of the participant’s preference upon their acceptance. The snowball technique was sequentially adopted^(15)^.

A total of 24 nurses were interviewed, with no sample loss, and the data saturation criterion was used to end the interviews. This means that no new elements were identified in the interviews and there was no need to add new information, as this does not alter the understanding of the phenomenon under scrutiny^(16)^.

### Data collection and organization

Data collection took place from January to February 2022. An interview was conducted using a semi-structured script, developed and applied by the researchers themselves after three pilot tests, carried out at the institution itself and adjusted as needed.

The script consisted of two parts, namely: characterization of the participant (identification code, gender, age, length of service and sector/unit assigned) and three guiding questions, namely: “Can you tell me what difficulties you had in providing physical care to patients with COVID-19?”, “Can you tell me what difficulties you had in providing psychosocial care to patients with COVID-19?” and, “Can you tell me what difficulties you faced in providing relational care to patients with COVID-19?”. The questions were accompanied by a vignette to provide a conceptual explanation to the participant in the care, regarding the chosen theoretical framework.

The interviews were conducted by two master’s students and a PhD student. To ensure the accuracy of data collection, the researchers received training in a research group. The interviews were recorded with their own smartphone device. The interviews had an average duration of 20 minutes. The recorded interviews were later transcribed in full in a Microsoft Office Word document^®^.

### Data Analysis

For the analysis, the content analysis technique proposed by Creswell was followed, namely: pre-analysis; exploration of the material or coding and treatment of the results and interpretation^(17)^. The data were grouped and regrouped, extracting fragments of greater relevance, with compacted information. For this purpose, the MaxQda^®^ Analytics Pro 22 software was used, a tool that allowed grouping and quantification through the operationalization of the transcribed interviews. Three categories emerged that corresponded to the domains of FCs and their respective dimensions^(6)^.

## RESULTS

The average age of the 24 nurses was 41 years old. Most of them had worked for a minimum of three months and a maximum of two years in the hospital, and were mostly allocated to intensive care units for patients with COVID-19. Regarding gender, 71% of the participants were female and 29% were male.

### Physical care

The first category (i.e., Physical Care domain) encompassed aspects such as: Personal Hygiene and Dressings; Comfort and Mobilization; Eating and Drinking; Rest and Sleep; Safety and Medication Management.

In Personal Hygiene, the performance of bed baths in the form of a “dry bath” was scored, which is performed using moistened wipes without the use of soap and water. This occurred due to the severity of the patients’ clinical condition, in order to avoid hemodynamic decompensation of the patients.

[...] *there were patients who were significantly overweight and, when the situation was very serious, they were placed in a prone position, which made both oral and body hygiene difficult.* [...] *It was necessary to be very careful when performing dry baths; we did not wet the patient too much for fear of loosening a connection and causing contamination. This made our hygiene practice very difficult.* (Nure 5)

The measures taken in the Comfort and Mobilization dimension of care mainly involved the nurse’s assessment of the patient’s level of consciousness, respiratory rate, dyspnea, reported shortness of breath, and respiratory distress. For the nurses, there were situations in which pain control was difficult and, therefore, the comfort dimension was impaired. Due to the need for prone positioning and limited movement, pressure injuries (PIs) developed.

*The main difficulty of the patient with COVID is the lack of mobility due to dyspnea and respiratory problems, so we had a limitation in relation to all movements performed on the patient.* (Nurse 11)

[...] *the patient’s limited mobility also contributed to the development of injury.* (Nurse 2)

The Eating and Drinking care dimension was focused on those patients who would restart oral feeding after a speech therapy evaluation and on difficulties in aspects of administering a diet via a tube.

*Some strategies were created. Patients who had been intubated for a long time, when they underwent the tracheostomy procedure, began to be seen by the speech therapist. The professional sought to meet the patient’s needs. There were patients who wanted coffee, to taste it, or juice. We said: “instead of making this blue water, bring me some juice”.* (Nurse 6)

[...] *the way of measuring changed, we had to do total control, there were a lot of calculation errors in the balance* [water balance] [...]. (Nurse15)

There was a mention regarding COVID-19 patient care related to Rest and Sleep.

*We were unable to maintain the sleep of some patients due to the turnover and severity of the other patients who were next to them.* (Nurse 24)

The Safety dimension, in line with the theory, encompasses risk assessment and management, infection prevention and minimizing complications. This dimension of care can be understood from two perspectives: employee safety and patient safety.

*When providing COVID care, we used all the necessary equipment: gloves, masks, caps, face shields, glasses* [...]. (Nurse 1)

*In general, the team was very careful about hand hygiene, wearing protective equipment and taking safety precautions. I believe that due to fear of the disease, fear, and apprehension.* (Nurse 17)

[...] *for example, when transporting these patients to other sectors or for exams, we also started to organize ourselves better* [...]. (Nurse 4)

[...] *the* [venous] *accesses were not always checked, due to the demand, the flow of patients was very high* [...] *physical care was often neglected.* (Nurse 24)

Regarding the Medication Management dimension, the difficulties were related to the initial medication shortage and difficulties in dilution/administration, which led to it being considered a priority care activity.

*A priority for these more serious patients, as was the case with COVID, was care related to vasoactive drugs and sedation, maintaining a flow* [...] *not letting it run out* [...] *because the flow was very high.* (Nurse 12)

*There were a lot of drugs that we weren’t used to using on a daily basis. Of course, the pharmacy gave us guidance, but a lot of things came in different arrangements, in different bottles* [...]. *We searched the internet to see how to dilute them correctly and do it in the best way, so as not to waste the medication. It was something that we, as nurses, were very concerned about, especially when they changed the dose, which was very different from the standard.* (Nurse 15)

### Psychosocial Care

The second category (Psychosocial Care domain) encompassed Communication (understood as verbal and non-verbal); Privacy; Dignity, Respect and Beliefs; and Emotional Well-being.

*The only contact he had was with our eyes.* (Nurse 9)

*Patients who were not intubated needed me to get feedback, there were no cell phones at the time, the unit had not yet had a cell phone, and I started making video calls with my cell phone.* (Nurse 23)

*Notes and nursing records were not a priority at the beginning of the greater demand, but the lack of them made it difficult to gather information and standardize it* [...]. (Nurse 18)

The Privacy, Dignity, Respect and Beliefs of the COVID-19 patient were also challenging when it involved aspects of the physical infrastructure, but as far as possible, we sought to achieve the aforementioned domain of care, mainly around spiritual aspects.

*It made me extremely uncomfortable to put a man and a woman in the same ward.* (Nurse 15)

[...] *I had to look at them a lot, say the same prayer, some belief* [...] *God or some kind of belief, we always took this precaution before intubating.* (Nurse 5)

*We talked to them while they slept. I talked to them about God, but I confess that I don’t know what their religion is, but I talked to them to trust in God, to believe.* (Nurse 13)

*We made little signs with all their preferences, what they like and what they don’t like, where they come from, who they live with, if they have pets.* (Nurse 14)

The care dimension Emotional Well-being was understood and responded to by participants from the perspective of their own well-being and not that of the patient, although important aspects were presented around the latter.

*We gave the option of video calls to the patient and family. So, this was also a comfort, both for the family and for us, as it minimized the longing and worry.* (Nurse 8)

*There were people who worked, reached a certain point and couldn’t take it anymore.* (Nurse 2)

[...] *I think I ‘rescued myself’ with a nurse there during the time I worked with COVID.* (Nurse 8)

### Relational care

The third category (Relational Care domain) encompassed Active Listening; Empathy and Compassion; Engagement, Support and Involvement of Families and Caregivers; working with patients to define, achieve and evaluate progression of goals.

The Active Listening dimension was presented to both the family member and the patient.

[...] *Many told you that he couldn’t die... and that he wanted to talk to his family, see his grandson* [...]. *He could be intubated, but nothing bad could happen to him.* (Nurse 3)

*It wasn’t just a time to check vital signs and administer medication, but also a time for active listening. It was certainly there, at that moment, that we knew what needed to be done.* (Nurse 19)

The dimensions Empathy and Compassion and Engagement with Patients occurred during the work routine. The weaknesses were perceived:

*We put a patient identification in each box, as if it were a personal file: ‘So, who am I? I’m married, I have children* [...]*. An emotional record* [...]. *What do I do for a living? What do I like? I like music; I saw in the emotional record that the patient liked rock, so sometimes I put it on my cell phone, on Spotify’.* (Nurse 6)

*There were cases where there were pregnant women here, and it was very difficult to deal with the situation, the father wanting to know how his child was doing and you having to say that, in the future, you might have to choose between the child and the mother. It was difficult to hear that and not put yourself in the other person’s shoes.* (Nurse 24)

*Sometimes I even saw the funeral home’s lack of preparation with that body; it was as if you were nothing. It was very sad!* (Nurse 3)

[...] *that patient, unfortunately, died alone.* (Nurse 7)

The dimension Support and Involvement of Families and Caregivers was highlighted, within the possibilities of nursing. This, in turn, implied the dimension of Helping Patients to remain calm.

[...] *the family’s autonomy was respected. We passed on the information over the phone or tried to interact in some way to alleviate fears. We talked to the family members outside* [the hospital]*, explaining everything that was being done and how the disease was progressing.* (Nurse 17)

[...] *when a patient with COVID was admitted, he or she already had significant fear due to the media coverage. There were psychologists who offered support to the patients, but the nurses worked more on a day-to-day basis, providing reassurance.* (Nurse 11)

[...] *I think that the nurses can always find a way to look at a patient who is looking in pain. Sometimes, all it takes is holding the patient’s hand or stroking their hair.* (Nurse 18)

The aspect related to working with patients aims to define, achieve and evaluate the progression of goals. In the meantime, video calls were mentioned as a tool for humanizing care and bonding with the patient and their caregivers.

*Sometimes, this video call changes the patient’s appearance and the entire context as well: how to approach the family member, because you are getting to know them and you also know how to better deal with the patient.* (Nurse 4)

## DISCUSSION

Nursing played a leading role in confronting the COVID-19 pandemic. Nurses used previous experiences and strategies that are inseparable from management/care to mitigate the impacts and meet the demand arising from the pandemic^(12)^.

In terms of care, the FC presents a structure that involves nurses’ actions in response to the person’s essential needs and, given the results, nursing was perceived as a category closely linked to these practices. Body and oral/buccal hygiene, in the Personal Hygiene (Physical Care) dimension, was highlighted by an international mixed-method study using FCs in patients with COVID-19. In it, patients’ personal care was lost or delayed, mainly in relation to oral/buccal hygiene, but also with washing, brushing and using the bathroom. This time was spent less, but they were still encouraged to do it^(5)^.

Based on the findings of this study, it was noted that bathing the patient in bed was difficult and therefore took longer to perform. There was no mention of specific scalp hygiene care, which may be due to the nursing culture of understanding bathing as a cephalocaudal intervention. Furthermore, there was no mention of cleaning the environment, which can be explained by the understanding of nursing assistants, who are generally assigned a management role.

It is considered that Physical Care is an opportunity for nurses to get closer to the patient. For example, when nurses perform bathing, even if not in the usual way, the FC is occurring^(6)^. In light of the FC, it can be inferred that delegating or even failing to perform the procedure may be a factor that would weaken their technical work with the patient^(6-8)^. Thus, although there are dentists and physiotherapists in the hospital environment, especially in relation to patients under intensive care, who also participate in oral/mouth hygiene, nursing was linked to this care.

The difficulty in performing personal hygiene and dressings could be related to the use of vasoactive drugs by the patient, which, in turn, could cause hemodynamic instability. The preparation, dilution and administration of drugs, mainly vasoactive, sedative and antibiotic drugs, according to reports, were the care priorities. Contrary to this data, the same previous international study^(5)^ indicated that some professionals were unable to double-check the medications, which caused an increase in errors, delays and lack of pharmaceutical support. The data from this research indicate that support from this in the institution is weak due to lack of knowledge of dosages, prescription demand and drug shortages at times.

Given all the demands and care for the critical profile of patients^(11)^, it was noted that feeding, comfort, sleep and rest, that is, aspects that also concern fundamental care for hospitalized patients(6-8), were compromised. Prone positioning was a challenge, in addition to interrupting sleep or even making it impossible to provide peaceful sleep without noise^(5)^. In contrast to this study, the findings of this research do not point to problems with equipment, and the main focus was on tube feeding for intubated patients. A Polish observational study found that the main difficulty in ensuring adequate nutritional support was attributed to the need for ventilation in the prone position, which reinforces the importance of multidisciplinary discussion in order to comply with nutritional recommendations in this clinical scenario^(18)^.

Such care is an essential part of Brazilian nursing, including being included in the psychobiological category of Basic Human Needs of the individual, often cited in curricula. This is a theoretical framework proposed by Wanda de Aguiar Horta, which aims to meet human needs in the health-illness cycle at any stage of life and, when not met, causes hemodynamic instability in the life cycle^(19)^.

A study conducted in the state of Minas Gerais, Brazil, indicated the bedside care model as a stimulus for innovative practice, (re)directing nurses in the search for their telos, patient care, overcoming the fragmentation of nursing care and reconfiguring professional identity^(20)^. In the same study, the model in the scenario brought up challenges related to the recognition of nurses by the multidisciplinary team, generating identity ruptures^(20)^.

In Psychosocial Care^(7,8)^, the communication dimension proved to be a relevant aspect in the nurse-patient interface. Corroborating another study, although not in a hospital setting, the use of WhatsApp was understood as a powerhouse for establishing communication, especially between the family and the healthcare team^(21)^. During the pandemic period, there was an effort to synchronize communication between all professionals so that information could be passed on to the family member^(22)^.

Although mentioned, it was noted that Psychosocial Care was less neglected by nursing in relation to Physical Care. In part, this factor is due to the fear and emotional suffering that nursing itself experienced. During the pandemic, there was a weakness in providing normal levels of support by nurses, for example, skin-to-skin touch, time for communication and listening^(5)^.

Belief was a matter of concern for the professionals in this study, contradicting another study that showed a lack of knowledge about patients’ beliefs, in addition to the absence of support from the hospital chaplaincy^(5)^. However, during a pandemic, feelings and emotions related to faith and spirituality were commonplace and should be considered, as they revealed what was experienced, which can be understood with a careful and sensitive eye^(21)^.

The loss of people in a short space of time, the difficulties in carrying out farewell rituals between people who are about to die, as well as funeral rituals, can make the experience of mourning difficult. As a result, expressions of affection, condolences and spirituality have undergone changes, and thus, the importance of promoting alternative and respectful ways to ritualize the processes experienced is discussed, which seemed essential to resignify losses and face challenges during and after the pandemic^(23)^.

Research conducted worldwide describes that frontline nurses experienced an enormous workload; long-term fatigue; threat of infection; and anxieties and frustrations regarding the death of patients they cared for. In addition, they worried about their families and vice versa^(24)^. In this dimension, it is important that workers rest adequately and that their critical personal needs and emotional support are met, thus contributing to maintaining individual and team performance in the long term^(25)^, in addition to indirectly reflecting on the provision of FC.

FC is at the core of nursing practice. It reflects nursing values and highlights the primacy of being with, doing and authentically engaging with patients and those who support them, and encompasses the most intimate personal needs, shedding light on Relational Care^(8)^. In this sense, empathy and compassion, being engaged with patients’ needs, being “with” and “for” the patient and active listening can make a difference for the patient and their health needs^(7,8)^.

Patients lost visits from other important people and were isolated. Staff had fewer opportunities to build relationships with important people and manage their emotional needs. Staff had difficulty keeping loved ones fully informed by telephone while ensuring that confidentiality was maintained^(5)^. Similar to the findings of this study, in which nurses managed to engage with patients and provide support to family members and caregivers, in a limited way and within their possibilities.

In nursing, there is much talk in the area about placing patients in the position of protagonists of their own care. However, it was noted that little was possible in practice during the pandemic, at least in the hospital environment. This may be related to the profile of critical patients in the sectors, which requires rapid decision-making guided by policies and protocols, with a little less involvement from the patient and other important people^(1-5)^. It can be interpreted that the goal of the nursing team was to not cause harm to the patient in the long term, as a positive consequence of their prioritized care.

In view of the whole, FC becomes present in the care provided to COVID patients by nursing. However, it is inferred that it would be necessary to conduct this study in other contexts. For example, a cross-sectional study conducted in five countries found that nursing students are not correctly identifying all of a patient’s fundamental care needs when faced with different care scenarios. The highest frequency of identification was for physical care needs and the lowest for psychosocial and relational needs, corroborating a general analysis of this research^(26)^.

The prominence and importance of leadership in FC has never been more pertinent than in the past 2 years, with the advent of the global COVID-19 pandemic and the need to respond to unprecedented health and nursing care demands^(27)^. It is therefore suggested that using the COVID pandemic crisis as an opportunity for transformational change in the delivery of fundamental care may provide some answers^(28)^.

### Study limitations

It is considered that the main limitation was the study scenario being reduced to just one context, a single hospital. However, in an adverse situation, the professionals in this scenario, even though sensitized, collaborated to assist patients within a prioritization of possible care, which demands such importance.

### Contributions to the field of nursing

It is proposed that the main contributions of this study to the area are the urgency of nurses returning to interacting directly with patients, bedside nursing, and not just focusing their work process on managing care. The pandemic period may have provided this opportunity or even raised the relevance of the debate by the category. The relevance of the theory, little known in the national scenario, also constitutes an important foundation for nursing as a discipline and in the resumption of its telos.

## FINAL CONSIDERATIONS

Given the results and in response to the proposed objective, it is possible to verify that fundamental care was present in the actions of the nurse during the care of COVID-19. Within their limitations and difficulties in the current situation, the nurse (re)approached the patient in their care, in addition to managing the care.

Although with weaknesses, in physical care, aspects such as safety, hemodynamic instability, devices, dilution and high-volume medication administration implied that FC were overlooked in favor of technical care. It is noteworthy that, although the care was limited, other fundamental care was extremely explored, due to the sensitivity generated, such as relational care, respect for beliefs and sensitivity to the situation of isolation and death. Nurses’ personal experiences brought them closer to patients and family members.
