# Supplementary material for: Nursing care for hospitalized patients with COVID-19 in light of Fundamental Care
Source: Rev Bras Enferm. 2025 Apr 28;78(2):e20240075. doi: 10.1590/0034-7167-2024-0075 (PMC12037185; doi:10.1590/0034-7167-2024-0075)
Supplement: Translation

# Translation: Assistência de enfermagem ao paciente hospitalizado com COVID-19 à luz do Cuidado Fundamental

**DOI:** 10.1590/0034-7167-2024-0075pt

**Authors:** Fabieli Borges, Elizabeth Bernardino, Camila Rorato, Daniele Cristina dos Reis Bobrowec, Olívia Luciana dos Santos Silva, Amanda Gomes Ribeiro Pujol de Carvalho, Clémence Dallaire

**Affiliations:** I Universidade Federal do Paraná. Curitiba, Paraná, Brasil; II Université Laval. Quebec, Canadá

**Keywords:** Assistência de Enfermagem, Cuidado, COVID-19, Paciente, Hospital

## Abstract

**Objetivos:**

analisar como a pandemia de COVID-19 implicou no Cuidado Fundamental prestado por enfermeiros ao paciente hospitalizado em um hospital público.

**Métodos:**

pesquisa qualitativa, descritiva, exploratória. Foram entrevistados 24 enfermeiros que assistiram a pacientes com COVID-19 em um hospital público na capital no Paraná, de janeiro a fevereiro de 2022. Aplicou-se aos dados a Análise de Conteúdo de Creswell, operacionalizados pelo *software* MaxQda e à luz da teoria dos Cuidados Fundamentais.

**Resultados:**

foi obtido três categorias com suas dimensões respectivas: Cuidados Físicos (Higiene Pessoal; Conforto e Mobilização; Comer e beber; Descanso e sono; Segurança e Gerenciamento de medicamentos), Cuidado Psicossocial (Comunicação; Privacidade; dignidade, respeito e crenças; bem-estar emocional) e Cuidado Relacional (Escuta ativa; Empatia e compaixão; Engajamento, apoio e envolvimento a famílias e cuidadores e, trabalho com os pacientes).

**Considerações Finais:**

o período da pandemia pode ter oportunizado o olhar na relação enfermeiro-paciente-assistir especialmente alicerçado na teoria dos Cuidados Fundamentais.

## INTRODUÇÃO

A pandemia de COVID-19 apresentou um desafio sem precedentes à população mundial e à saúde pública, mesmo em países de alta renda^(1,2)^. Inseridos na Rede de Atenção à Saúde (RAS), os hospitais de ensino foram requisitados pelos gestores para ofertar leitos de UTI e de enfermarias especializadas, de modo que esses espaços tiveram que, rapidamente, realizar a gestão do cuidado estruturalmente e em estratégias para o enfrentamento à COVID-19^(1)^.

A assistência aos pacientes com COVID-19, em decorrência da necessidade premente de leitos hospitalares, tornou o escalonamento das condições clínicas do paciente um critério essencial para o melhor aproveitamento das unidades assistenciais que compõem a RAS^(3)^. Pelo que se consegue apreender nos dias atuais e na observação empírica mostrada cotidianamente acerca dos cenários de saúde, é possível refletir que o paciente, para além da questão clínica imposta pelo vírus, pode ter sido afetado em outros aspectos da sua condição de saúde^(4,5)^.

Especificamente durante sua hospitalização, cabe observar uma possível (des)assistência da enfermagem para aspectos relacionados aos cuidados oferecidos aos pacientes. Nesse interim, um referencial teórico que sustenta o rol de cuidados proposto por Kitson e demais autores, há pouco mais de uma década, isto é, o denominado Cuidado Fundamental (CF)^(6)^, pode ser utilizado para lançar luz ao cuidado à época. Conceitualmente, o CF diz respeito a uma estrutura de cuidado holística e centrada no paciente, englobando ações de enfermagem que respeitam e se concentram nas necessidades essenciais de uma pessoa para garantir sua integridade física e bem-estar psicossocial. Tais necessidades são satisfeitas por meio do desenvolvimento de uma relação positiva e de confiança com a pessoa a ser cuidada, bem como com a sua família e cuidadores^(7,8)^.

Prezando justamente pelo cuidado, seja na assistência direta, seja nas ações da gestão, os hospitais públicos de ensino e federais buscaram alcançar a sua responsabilidade social no enfrentamento da pandemia. Um estudo documental realizado com 44 hospitais universitários da rede federal de ensino do Brasil apontou, no primeiro ano da pandemia, um total de 495 ações para o enfrentamento da pandemia de COVID-19, as quais se distribuíram entre a Assistência (38,99%), Gestão (37,58%), Extensão (16,16%) e Ensino e Pesquisa (7,27%)^(9)^. Sendo a assistência aquela que compõe uma proporção maior, a relevância desta pesquisa consiste em conhecer como ocorreu a oferta de cuidados fundamentais em meio à demanda vivenciada.

Com toda estrutura e tecnologia disponíveis, usualmente, os pacientes referenciados aos hospitais foram aqueles mais críticos e dependentes de cuidados considerados complexos^(1,10)^. Embora tenha sido um desafio no que se refere à regulação de leitos e critérios de admissão do paciente portador de COVID-19, quando se tinha uma oferta menor que a demanda, esta era somada às de filas de espera, que já se apresentavam como um problema anterior^(11-13)^.

O perfil de pacientes exigiu uma (re)orientação na assistência prestada pela equipe de enfermagem à beira leito, sobretudo no que concerne aos Cuidados Fundamentais (CFs). Sob essa premissa, apresenta-se a seguinte questão norteadora: como a pandemia de COVID-19 implicou na prestação de cuidados fundamentais realizado por enfermeiros em hospital público de ensino?

## OBJETIVOS

Analisar como a pandemia de COVID-19 implicou no Cuidado Fundamental prestado por enfermeiros ao paciente hospitalizado em um hospital universitário.

## MÉTODOS

### Aspectos éticos

Este estudo foi aprovado pelo Comitê de Ética do Complexo Hospital de Clínicas da Universidade Federal do Paraná. Respeitaram-se a Resolução do Conselho Nacional de Saúde n° 466, de 12 de dezembro de 2012. O Termo de Consentimento Livre e Esclarecido (TCLE) foi apresentado e obtido mediante assinatura escrita de todos os participantes. Ademais, em respeito ao anonimato dos participantes, utilizou-se da codificação: enfermeiro (Enf), seguido da ordem numérica, Ex. Enf1, Enf2 e assim sucessivamente.

### Referencial teórico-metodológico

Este estudo ancora-se no referencial teórico de Cuidado Fundamental (CF) proposto por Kitson e demais autores^(6)^. O Cuidado Fundamental apresenta uma estrutura que envolve ações do enfermeiro face às necessidades essenciais da pessoa. Pode-se dizer, então, que os cuidados fundamentais abrangem atividades de Cuidados Físicos, tais como a higiene pessoal, conforto, descanso e sono, Cuidados Psicossociais, abrangendo a privacidade, dignidade, cuidado emocional e interação social e Cuidados Relacionais, como a empatia, compaixão e apoio^(7,8)^.

### Tipo de estudo

Trata-se de uma pesquisa qualitativa, descritiva e exploratória. Obedeceu-se aos critérios apresentados no *Consolidated Criteria for Reporting Qualitative Research* (COREQ)^(14)^.

### Cenário do estudo

A pesquisa foi realizada em um hospital público de ensino situado na capital do Paraná. O hospital é referência para pacientes de todo o estado, oferecendo tratamento ambulatorial, clínico e cirúrgico, e dispõe de cerca de 424 leitos. No decorrer da pandemia, o hospital ampliou sua capacidade com a adição de 89 leitos de enfermaria e 78 leitos de terapia intensiva.

### Fonte dos dados

Foram incluídos enfermeiros que atuaram diretamente na assistência a pacientes durante a pandemia COVID-19, no período diurno e noturno. O não comparecimento à entrevista após três agendamentos acordado com o pesquisador, bem como o período de férias, licença maternidade dos profissionais foram considerados critérios de exclusão para a pesquisa. A amostra foi intencional de modo a proceder escolha de representantes-chave que atuaram durante a pandemia na linha de frente. Foi contatado inicialmente a coordenação de enfermagem da unidade de infectologia, setor central para o atendimento à demanda da pandemia COVID-19 na instituição. Os objetivos do estudo foram explanados e procedeu-se ao agendamento da entrevista no local, data e horário de preferência do participante mediante o seu aceite. Adotou-se sequencialmente, a técnica bola de neve^(15)^.

Totalizaram-se 24 enfermeiros, sem perda amostral e o critério de saturação dos dados foi utilizado para o encerramento das entrevistas. Isso significa que nenhum novo elemento foi identificado nas entrevistas e não houve necessidade de acrescentar novas informações, pois isso não altera a compreensão do fenômeno em questão^(16)^.

### Coleta e organização dos dados

A coleta de dados ocorreu no período de janeiro a fevereiro de 2022. Realizou-se uma entrevista com o subsídio do roteiro semiestruturado, elaborado e aplicado pelas próprias pesquisadoras após três testes-piloto, feito na própria instituição e ajustado conforme necessidade.

O roteiro foi composto por duas partes, a saber: caracterização do participante (Código identificador, Sexo, Idade, Tempo de atuação e setor/unidade lotada) e três questões norteadoras, a saber: “Pode me dizer quais foram as suas dificuldades para realizar os cuidados físicos dos pacientes com COVID-19?”, “Pode me dizer quais foram as suas dificuldades para realizar os cuidados psicossociais dos pacientes com COVID-19?” e, “Pode me dizer quais foram as suas dificuldades para realizar os cuidados relacionais dos pacientes com COVID-19?”. As questões foram acompanhadas por uma vinheta para explanação conceitual ao participante em atendimento, quanto ao referencial teórico escolhido.

As entrevistas foram conduzidas por duas mestrandas e uma doutoranda. Para garantir a precisão da coleta de dados, as pesquisadoras receberam treinamento em grupo de pesquisa. As entrevistas foram gravadas com dispositivo *smartphone* próprio, totalizando uma média de 20 minutos de duração. Posteriormente, as entrevistas gravadas foram transcritas na íntegra no documento *Microsoft Office Word* ^®^.

### Análise dos dados

Para a análise, obedeceu-se à técnica de análise de conteúdo proposta por Creswell, quais sejam: pré-análise; exploração do material ou codificação e tratamento dos resultados e interpretação^(17)^. Os dados foram agrupados e reagrupados, extraindo-se fragmentos de maior relevância, com informações compactadas. Para tanto, utilizou-se o *software* MaxQda^®^ Analytics Pro 22, ferramenta que permitiu agrupar e quantificar por meio da operacionalização das entrevistas transcritas. Emergiram três categorias que corresponderam aos domínios de CFs e suas respectivas dimensões^(6)^.

## RESULTADOS

O total de 24 enfermeiros tinham, em média, 41 anos de idade, eram atuantes com no mínimo três meses e no máximo dois anos no hospital, majoritariamente alocados em setores de cuidados intensivos a pacientes portadores de COVID-19. Referente ao sexo, 71% dos participantes foram do sexo feminino e 29% do sexo masculino.

### Cuidados físicos

A primeira categoria (isto é, domínio Cuidados Físicos) englobou aspectos tais como: Higiene Pessoal e Curativos; Conforto e Mobilização; Comer e Beber; Descanso e Sono; Segurança e Gerenciamento de Medicamentos.

Na *Higiene Pessoal*, foi pontuada a realização do banho de leito na forma de “banho seco”, o que é realizado por meio de lenços umedecidos sem o uso de água e sabonete. Isso ocorreu em decorrência da gravidade do quadro clínico dos pacientes, a fim de evitar descompensação hemodinâmica dos pacientes.

[...] *havia pacientes com sobrepeso significativo e, quando a situação estava muito grave, eles eram colocados em posição prona, o que dificultava tanto a higiene oral quanto a higiene corporal.* [...] *Era necessário ter bastante cuidado ao fazer o banho seco; não molhávamos demais o paciente devido ao receio de soltar alguma conexão e causar contaminação. Isso dificultava bastante nossa prática de higienização.* (Enf5)

As medidas realizadas na dimensão de cuidado para o *Conforto e Mobilização* envolveram, principalmente, a avaliação pela enfermeira no nível de consciência do paciente, frequência respiratória, dispneia, falta de ar relatada e desconforto respiratório. Para os enfermeiros, houve situações em que o controle da dor era difícil e, assim, a dimensão conforto ficou prejudicada. Decorrente da necessidade do posicionamento em prona e da limitação de movimentos, houve o desenvolvimento de lesão por pressão (LP).

*A principal dificuldade do paciente com COVID é a falta de mobilidade por conta da dispneia, dos problemas respiratórios, então tínhamos uma limitação em relação a todo movimento realizado no paciente.* (Enf11)

[...] *a mobilidade limitada do paciente também contribuiu para o desenvolvimento de lesão.* (Enf2)

A dimensão de cuidado *Comer e Beber* foi centralizado naqueles pacientes que iriam reiniciar a alimentação por via oral após avaliação fonoaudiológica e nas dificuldades em aspectos da administração de dieta via sonda.

*Foram criadas algumas estratégias. Os pacientes que ficavam muito tempo entubados, quando realizavam o procedimento da traqueostomia já começavam a serem atendidos com a fonoaudióloga. A profissional buscava atender a necessidade do paciente. Tinha paciente que queria café, para sentir o gosto, queria suco. Falávamos: “em vez de você fazer essa água azul, traz um suco”.* (Enf6)

[...] *mudou a forma de mensurar, tinha que fazer o controle total, dava muito erro de cálculo no balanço* [balanço hídrico] [...]. (Enf15)

Houve uma menção em relação ao cuidado ao paciente COVID-19 relacionado ao *Descanso* e *Sono*.

*Não conseguíamos manter o sono preservado de alguns pacientes devido à rotatividade e à gravidade dos outros pacientes que estavam ao lado.* (Enf24)

A dimensão de *Segurança*, em consonância à teoria, engloba a avaliação e gerenciamento de riscos, prevenção de infecções e minimização de complicações. Essa dimensão de cuidado pode ser compreendida por dois prismas: a segurança dos funcionários e a segurança do paciente.

*No atendimento da COVID usávamos toda a paramentação: luva, máscara, touca, face-shield, óculos* [...]. (Enf1)

*No geral, a equipe era bem cuidadosa com a realização da higienização das mãos, a paramentação e os cuidados de segurança. Creio que pelo temor à doença, o medo, o receio.* (Enf17)

[...] *por exemplo, o transporte desses pacientes para outros setores ou para fazer exames, nós também começamos a nos organizar melhor* [...]. (Enf4)

[...] *os acessos* [venosos] *nem sempre eram revisados, por conta da demanda, o fluxo era muito grande de pacientes* [...] *os cuidados físicos muitas vezes foram negligenciados sim.* (Enf24)

No tocante à dimensão de *Gerenciamento de Medicamentos*, as dificuldades estiveram relacionadas à escassez medicamentosa inicial e às dificuldades na diluição/administração, o que levou a ser considerado uma atividade prioritária de cuidado.

*Uma prioridade para esses pacientes mais graves, como era o caso da COVID, era o atendimento relacionado às drogas vasoativas e às sedações, mantendo uma vazão* [...] *não deixar acabar* [...] *porque a vazão era muito alta.* (Enf12)

*Muita droga que a gente não estava acostumada a usar no dia a dia. É claro que a farmácia nos orientava, mas muita coisa vinha em disposição diferente, em frasco diferente* [...]*. A gente procurava na internet para ver qual era a forma de diluir corretamente e fazer da melhor maneira, para não perder a medicação. Era algo com o qual a gente, como enfermeiro, se preocupava bastante, especialmente quando mudavam a dose, que era muito diferente do padrão.* (Enf15)

### Cuidado Psicossocial

A segunda categoria (domínio de Cuidado Psicossocial), englobou a Comunicação (entendida como verbal e não verbal); Privacidade; Dignidade, Respeito e Crenças e Bem-estar emocional.

A Comunicação enfermeiro-paciente e paciente-familiar ocorreu de forma verbal e não-verbal, porém, limitada.

*O único contato que ele tinha era com os nossos olhares.* (Enf9)

*Os pacientes que não estavam entubados eles precisavam de mim para ter um retorno, não tinha celular na época não tinha sido instituído o celular da unidade e eu comecei a fazer as videochamadas com o meu celular.* (Enf23)

*As anotações, registros de enfermagem não foram uma prioridade no início de maior demanda, mas a falta dela foi uma dificuldade para reunir informações e padronizá-las* [...]. (Enf18)

A *Privacidade, a Dignidade, o Respeito e as Crenças* do paciente com COVID-19 também foram desafiadoras quando envolvia aspectos da infraestrutura física, mas dentro do possível, buscou-se alcançar o referido domínio de cuidado, principalmente em torno de aspectos espirituais.

*Me deixou extremamente desconfortável colocar um homem e uma mulher na mesma enfermaria.* (Enf15)

*[...] Eu tinha bastante o olhar, fazer a mesma oração, alguma crença* [...] *Deus ou algum tipo de crença a gente sempre teve este cuidado antes de intubar.* (Enf5)

*Conversávamos com eles enquanto dormiam. Eu conversava com eles de Deus, mas assim, confesso que eu não sei qual a religião deles, mas eu conversava para confiar em Deus, acreditar.* (Enf13)

*Fazíamos plaquinhas com todas as preferências, o que gosta e o que não gosta, de onde vem, com quem mora, se tem animal de estimação.* (Enf14)

A dimensão de cuidado Bem-estar emocional foi entendida e respondida pelos participantes na perspectiva do seu próprio bem-estar e não a do paciente, embora tenha-se apresentado aspectos importantes em torno deste último.

*Dávamos a opção de vídeo-chamada para o paciente e família. Então, isso também era um acalento, tanto para a família quanto para nós, pois, minimizava a saudade e a preocupação.* (Enf8)

*Teve gente que trabalhou, chegou a um determinado momento e não aguentou mais.* (Enf2)

[...] *eu acho que eu ‘me resgatei’ com uma enfermeira ali nesse tempo em que trabalhei com COVID.* (Enf8)

### Cuidado Relacional

A terceira categoria (domínio Cuidado Relacional), englobou a Escuta Ativa; Empatia e Compaixão; Engajamento, Apoio e Envolvimento a Famílias e Cuidadores; trabalho com os pacientes para definir, alcançar e avaliar progressão de metas.

A dimensão *Escuta Ativa* foi apresentada tanto ao familiar quanto ao paciente.

[...] *Muitos relatavam para você que ele não podia morrer... e que ele queria falar com a família, ver o neto* [...]. *Ele poderia ser entubado, mas não era para deixar acontecer nada de ruim com ele.* (Enf3)

*Não era apenas um momento de checagem de sinais vitais e administração de medicação, mas também um momento de escuta ativa. Com certeza, era ali que, naquele instante, sabíamos o que precisava ser feito.* (Enf19)

As dimensões *Empatia e Compaixão* e *Engajamento com Pacientes*, ocorreram durante a rotina de trabalho. As fragilidades foram percebidas:

*Colocávamos em cada box uma identificação do paciente, como se fosse uma ficha pessoal: ‘Então, quem eu sou? Sou casado, tenho filhos* [...]*. Um prontuário afetivo [...]. Eu trabalho com o quê? O que eu gosto? Eu gosto de música; eu via no prontuário afetivo que o paciente gostava de rock, então às vezes eu colocava no meu celular, no Spotify’.* (Enf6)

Teve casos em que havia gestantes aqui, e era bem difícil lidar com a situação, o pai querendo saber como estava o filho e você tendo que dizer que, futuramente, poderia ter que escolher entre o filho e a mãe. Era difícil ouvir isso e não se colocar no lugar do outro. (Enf24)

*Eu via, às vezes, até o despreparo da funerária com aquele corpo; era como se você se resumisse a nada. Foi muito triste!* (Enf3)

*[...] aquela paciente, infelizmente, faleceu sozinha.* (Enf7)

Foi evidenciado a dimensão *Apoio e Envolvimento a Famílias e Cuidadores*, dentro da possiblidade pela enfermagem. Que, por sua vez, implicava na dimensão de *Ajuda aos Pacientes* para manter a calma.

[...] *respeitava-se autonomia da família. Passávamos os dados por telefone ou tentávamos interagir de alguma forma para amenizar medos. Conversávamos com os familiares do lado de fora* [do hospital]*, explicando tudo o que estava sendo feito e como estava a progressão da doença.* (Enf17)

[...] *quando um paciente com COVID era internado, ele já apresentava um medo significativo devido à divulgação pelas mídias. Havia psicólogos que ofereciam apoio aos pacientes, mas a enfermagem atuava mais no dia a dia, passando segurança.* (Enf11)

[...] *eu acho que a enfermagem sempre consegue encontrar uma forma de olhar para aquele paciente que está com uma expressão de dor. Às vezes, é só segurar a mão do paciente ou passar a mão no cabelo.* (Enf18)

O aspecto relacionado a trabalhar com pacientes tem por objetivo a definição, alcance e avaliação da progressão de metas. Nesse ínterim, as vídeo-chamadas foram mencionadas como uma ferramenta de humanização do cuidado e de vinculação com o paciente e seus cuidadores.

*Às vezes, essa vídeo-chamada muda o aspecto do paciente e todo o contexto também: como abordar o familiar, pois você está conhecendo-o e também tem como saber como lidar melhor com o paciente.* (Enf4)

## DISCUSSÃO

A enfermagem teve o seu protagonismo no enfrentamento à pandemia COVID-19. Os enfermeiros usaram de experiências anteriores e estratégias indissociáveis à gestão/assistência para mitigar os impactos e atender à demanda oriunda da pandemia^(12)^.

Assistencialmente, o CF apresenta uma estrutura que envolve ações do enfermeiro face às necessidades essenciais da pessoa e ante aos resultados, a enfermagem foi percebida como uma categoria com estreito vínculo a essas *práxis*. A higiene corporal e oral/bucal, na dimensão Higiene Pessoal (Cuidado Físico) foi apontada por um estudo de método misto internacional com uso dos CFs em pacientes com COVID-19. Nele, os cuidados pessoais dos pacientes foram perdidos ou atrasados, principalmente em relação à higiene oral/bucal, mas também com lavagens, escovação e uso do banheiro. Esse tempo foi gasto em tempo menor, mas que mesmo assim foram encorajados a fazê-lo^(5)^.

Frente aos achados desta pesquisa, percebeu-se que o banho no leito do paciente demandou dificuldade e assim, um tempo maior para a execução. Não houve menções em relação aos cuidados específicos de higiene de couro cabeludo, isso pode ser decorrente da cultura da enfermagem em entender o banho com intervenção cefálio-caudal. E, ainda, não houve menção à limpeza com o ambiente, que pode ser explicado pelo entendimento dos enfermeiros assistenciais, os quais geralmente são atribuídos como função gerencial.

Pondera-se que o Cuidado Físico é oportuno para a aproximação do enfermeiro e o paciente. Por exemplo, quando a enfermagem realiza o banho, mesmo que não da forma habitual, o CF está ocorrendo^(6)^. À luz do CF, pode-se inferir que delegar ou mesmo deixar de realizar o procedimento pode ser um fator que fragilizaria o seu fazer técnico junto ao paciente^(6-8)^. Assim, embora haja a presença de odontólogos e fisioterapeutas em ambiente hospitalar, sobretudo no que se refere a pacientes sob cuidados intensivos, os quais também participam da higiene oral/bucal, a enfermagem foi vinculada a esse cuidado.

A dificuldade na realização da higiene pessoal e dos curativos poderiam estar relacionados com o uso de drogas vasoativas pelo paciente que, por sua vez, poderiam causar uma instabilidade hemodinâmica. O preparo, diluição e administração das drogas, principalmente as vasoativas, sedativas e antibióticas, conforme relatos, foram as prioridades de cuidado. Contrário a este dado, o mesmo estudo internacional anterior^(5)^ apontou que alguns profissionais não conseguiram checar novamente os medicamentos, o que ocasionou aumento de erros, atrasos, e falta de apoio farmacêutico, os dados desta pesquisa apontam o apoio deste na instituição, com fragilidades frente ao desconhecimento de dosagens, demanda de prescrição e a escassez medicamentosa em alguns momentos.

Dada toda a demanda e cuidado com o perfil crítico dos pacientes^(11)^, percebeu-se que a alimentação, conforto, sono e descanso, isso é, aspectos que também dizem respeito ao cuidado fundamental para o paciente hospitalizado^(6-8)^, foram prejudicados. O decúbito ventral (‘proning’/pronação) foi um desafio, além da interrupção de sono ou mesmo a inviabilidade de oferta de sono tranquilo sem ruídos^(5)^. Em oposição a este estudo, os achados desta pesquisa não apontam problemas com equipamentos, e o destaque maior foi para a alimentação via sonda aos pacientes intubados. Um estudo observacional polonês constatou que a principal dificuldade para garantir suporte nutricional adequado foi atribuída à necessidade de ventilação em decúbito ventral, o que reforça a importância de discussão multiprofissional, de modo a cumprir as recomendações nutricionais nesse cenário clínico^(18)^.

Tais cuidados integram um cuidado essencial na enfermagem brasileira, inclusive disposta na categoria psicobiológica de Necessidades Humanas Básicas do indivíduo, tão citada em grades curriculares. Trata-se de um aporte teórico proposto por Wanda de Aguiar Horta, o qual objetiva o atendimento às necessidades humanas no ciclo saúde-enfermidade em qualquer fase da vida e, quando não atendidas, causam instabilidade hemodinâmica no ciclo vital^(19)^.

Uma pesquisa realizada no Estado de Minas Gerais, Brasil, apontou o modelo assistencial beira-leito como um estímulo à prática inovadora, (re)direcionando o enfermeiro na busca do seu *telos*, o cuidado ao paciente, superando a fragmentação da assistência de enfermagem e reconfigurando a identidade profissional^(20)^. No mesmo estudo, o modelo no cenário fez emergir desafios relacionados ao reconhecimento do enfermeiro pela equipe multiprofissional, gerando rupturas identitárias^(20)^.

Nos Cuidados Psicossociais^(7,8)^, a dimensão comunicação mostrou-se um aspecto relevante na interface enfermeiro-paciente. Corroborando com outro estudo, embora não numa realidade hospitalar, compreendeu-se o uso do *WhatsApp* como uma potência para o estabelecimento da comunicação, especialmente, entre a família e a equipe de saúde^(21)^. Houve, durante o período pandêmico, um esforço para sincronizar a comunicação entre todos os profissionais para que houvesse o repasse das informações ao familiar^(22)^.

Embora mencionados, percebeu-se que o Cuidado Psicossocial foi menos preterido para a enfermagem em relação ao Cuidado Físico. Em parte, tal fator incorre no medo e sofrimento emocional que a própria enfermagem vivenciou. Na pandemia, houve fragilidade em fornecer níveis normais de suporte pelos enfermeiros, por exemplo, toque pele a pele, tempo para comunicação e escuta^(5)^.

A crença foi pauta de preocupação pelos profissionais deste estudo, contradizendo outro estudo que mostrou a falta de conhecimento sobre as crenças dos pacientes, além da ausência de apoio da capelania no hospital^(5)^. No entanto, em um período pandêmico, os sentimentos e emoções relacionados à fé e espiritualidade foram cotidianos e devem ser considerados, pois revelaram o vivido, que pode ser compreendido por um olhar atento e sensível^(21)^.

As perdas de pessoas em curto espaço de tempo, as dificuldades para realização de rituais de despedida entre pessoas na iminência da morte de seus familiares, bem como de rituais funerários, podem dificultar a experiência do luto. Como resultado, expressões de afeto, condolências e espiritualidade sofreram alterações, e, assim, discute-se a importância de potencializar formas alternativas e respeitosas para ritualização dos processos vividos, o que pareceu essencial para ressignificar perdas e enfrentar desafios durante e após a vigência da pandemia^(23)^.

Pesquisas realizadas mundialmente descrevem que os enfermeiros da linha de frente passaram por uma carga de trabalho enorme; fadiga a longo prazo; ameaça de infecção; ansiedades e frustrações em relação à morte de pacientes de quem cuidavam. Além disso, eles se preocupavam com suas famílias e vice-versa^(24)^. Nessa dimensão, importa que os trabalhadores descansem adequadamente e que sejam atendidos nas suas necessidades pessoais críticas e apoio emocional, contribuindo, assim, para manter o desempenho individual e da equipe a longo prazo^(25)^, além de indiretamente refletir na prestação dos CFs.

O CF está no cerne da prática de enfermagem. Ele reflete os valores da enfermagem e destaca a primazia de *estar com,* fazer e se envolver autenticamente com os pacientes e aqueles que os apoiam, e abrange as necessidades pessoais mais íntimas lançando luz ao Cuidado Relacional^(8)^. Neste sentido, a empatia e compaixão, estar engajado com as necessidades dos pacientes, estar “com” e “para” o paciente e a escuta ativa podem ser um diferencial para o paciente e a sua necessidade de saúde^(7,8)^.

Os pacientes perderam as visitas de outras pessoas importantes e foram isolados. A equipe teve menos oportunidades de construir relacionamentos com pessoas importantes e administrar suas necessidades emocionais. Os funcionários tiveram dificuldades em manter os entes queridos totalmente informados por telefone, assegurando, ao mesmo tempo, que a confidencialidade fosse mantida^(5)^. De modo similar aos achados deste estudo, em que os enfermeiros conseguiram uma relação de engajamento com o paciente, apoio aos familiares e cuidadores, de forma limitada e dentro das suas possibilidades.

Na enfermagem, muito se é falado pela área para colocar o paciente na posição de protagonista do seu próprio cuidado. No entanto, percebeu-se que pouco foi possível na prática durante a pandemia, pelo menos, no que concerne ao ambiente hospitalar. Isso pode ter relação com o perfil de paciente crítico nos setores o qual requer tomada de decisão rápida e guiada por políticas, protocolos, com um pouco menos de envolvimento do paciente e de outras pessoas importantes^(1-5)^. Pode-se interpretar que a meta pela equipe de enfermagem era a de não causar malefício para o paciente a longo prazo, como uma consequência positiva do seu cuidado priorizado.

Frente ao todo, o CF torna-se presente nos atendimentos ao paciente COVID pela enfermagem. No entanto, infere-se que seria necessário realizar esse estudo em outros contextos. A título de exemplo, um estudo transversal realizado em cinco países constatou que estudantes de enfermagem não estão identificando corretamente todas as necessidades fundamentais de cuidado de um paciente quando se deparam com diferentes cenários de cuidado. A maior frequência de identificação se referiu às necessidades de cuidados físicos e a menos frequente para as necessidades psicossociais e relacionais, corroborando com uma análise geral desta pesquisa^(26)^.

A proeminência e a importância da liderança nos CFs nunca foram tão pertinentes como nos últimos 2 anos, com o advento da pandemia global da COVID-19 e a necessidade de responder a exigências de cuidados de saúde e de enfermagem sem precedentes^(27)^. Alvitra-se portanto, que aproveitar a crise pandêmica da COVID como uma oportunidade para uma mudança transformacional na prestação de cuidados fundamentais pode trazer algumas respostas^(28)^.

### Limitações do estudo

Considera-se que o principal limitador, foi o cenário do estudo reduzir-se a apenas uma realidade. No entanto, numa situação adversa, os profissionais deste cenário mesmo sensibilizados colaboraram para atender aos pacientes dentro de uma priorização de cuidados possíveis, o que demanda tamanha importância.

### Contribuições para a área da enfermagem

Propõe-se que as principais contribuições deste estudo para a área versam sob a urgência do enfermeiro voltar a se relacionar diretamente com o paciente, a enfermagem a beira-leito e não apenas centrar o seu processo de trabalho para o gerenciamento do cuidado. O período da pandemia pode ter oportunizado este olhar ou mesmo suscitado a pertinência do debate pela categoria. A pertinência da teoria, pouco conhecida no cenário nacional, também se configura um importante alicerce para a enfermagem enquanto disciplina e na retomada do seu *telos*.

## CONSIDERAÇÕES FINAIS

Diante dos resultados e em resposta ao objetivo proposto, é possível constatar que o cuidado fundamental esteve presente nas ações do enfermeiro durante o atendimento à COVID-19. Dentro de suas limitações e dificuldades face ao momento vivido, o enfermeiro (re)aproximou-se do paciente no seu assistir para além de gerenciar o cuidado.

Embora com fragilidades, no cuidado físico, aspectos como segurança, instabilidade hemodinâmica, dispositivos, diluição e administração medicamentosa em alto volume implicaram em CFs preteridos em favor dos cuidados técnicos. Ressalta-se que, embora os cuidados tenham sido limitados, outros cuidados fundamentais foram extremamente explorados, pela sensibilidade gerada como o cuidado relacional, respeito às crenças e à sensibilidade frente à situação de isolamento e morte. Experiências pessoais dos enfermeiros trouxeram mais aproximação com os pacientes e familiares.
